# Supplementary figures and images for: Bacterial diversity of cantaloupes and soil from Arizona and California commercial fields at the point of harvest
Source: PLoS One. 2024 Sep 26;19(9):e0307477. doi: 10.1371/journal.pone.0307477 (PMC11426484; doi:10.1371/journal.pone.0307477)

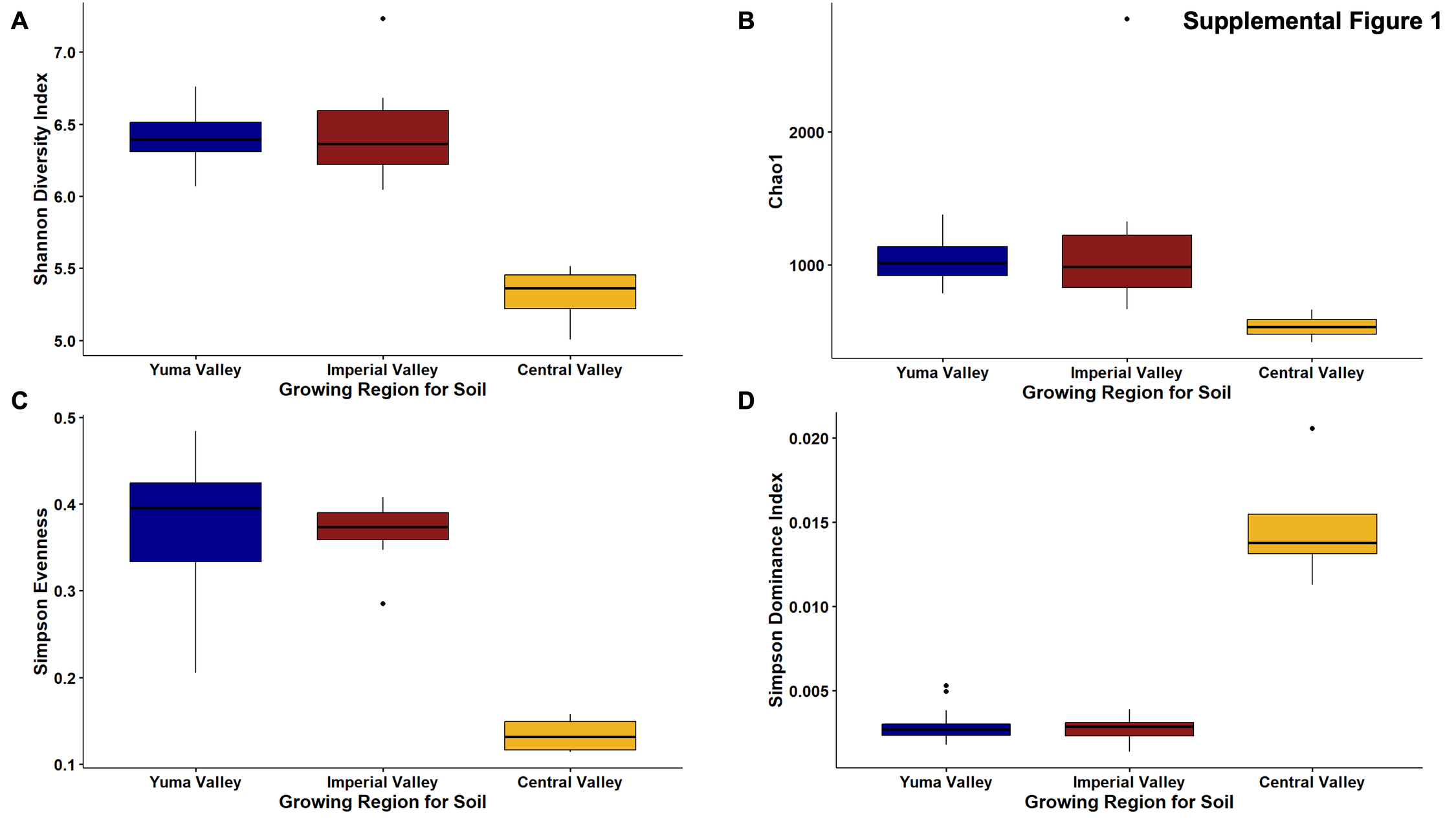

Supplement: S1 Fig — (A) Shannon Diversity Index plotted based on the site for the soil samples considering both richness and evenness. (B) Chao1 total richness plotted based on the site for the soil samples considering total taxonomic ASV richness. (C) Simpson Evenness plotted based on the site for the soil samples considering distribution (evenness) of the bacteria across the samples. (D) Simpson Dominance Index plotted based on the site for the soil samples considering inverse richness. (TIF) [file pone.0307477.s001.tif]

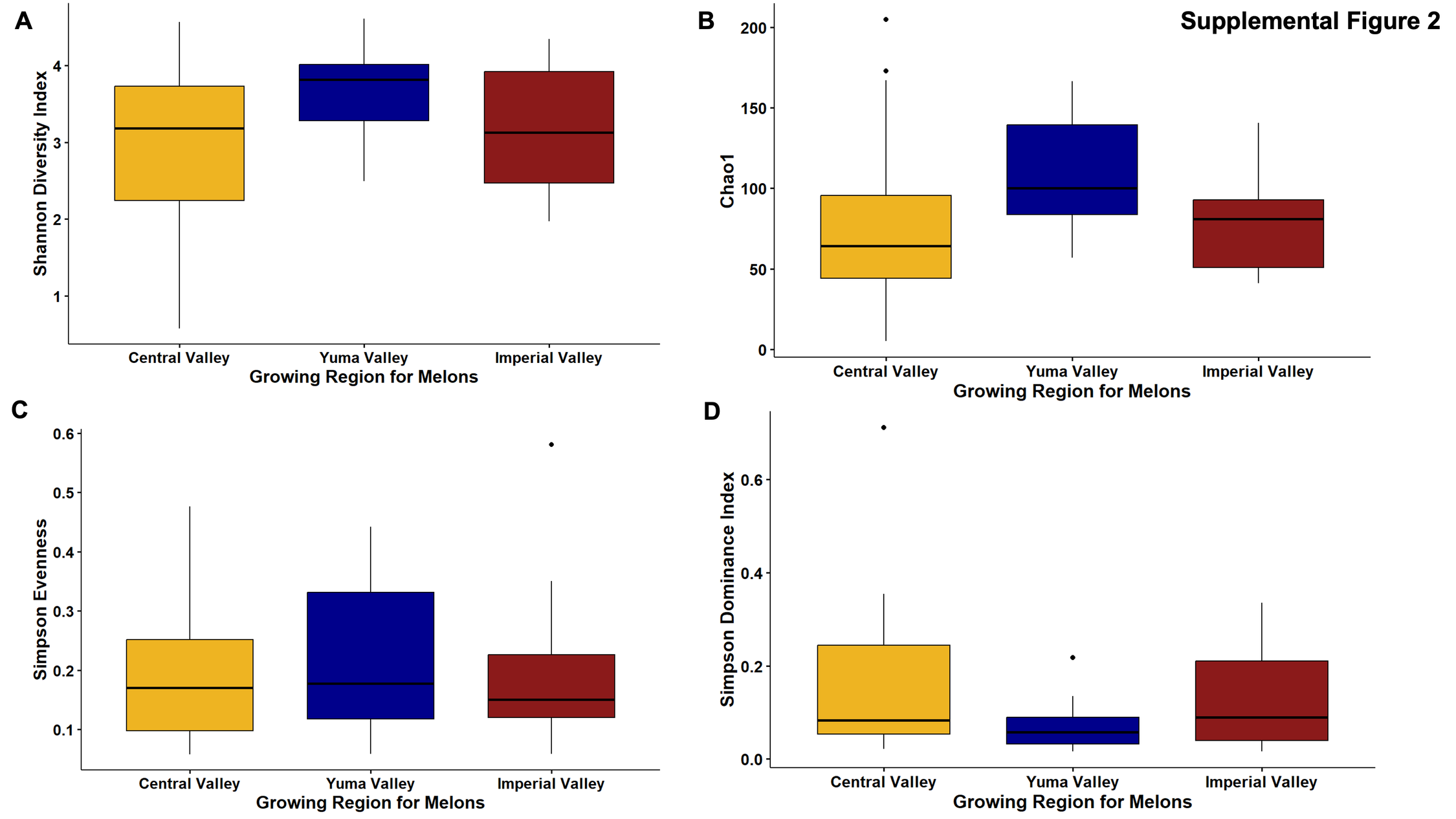

Supplement: S2 Fig — (A) Shannon Diversity Index plotted based on the site for the melon samples considering both richness and evenness. (B) Chao1 total richness plotted based on the site for the melon samples considering total taxonomic ASV richness. (C) Simpson Evenness plotted based on the site for the melon samples considering distribution (evenness) of the bacteria across the samples. (D) Simpson Dominance Index plotted based on the site for the melon samples considering inverse richness. (TIF) [file pone.0307477.s002.tif]

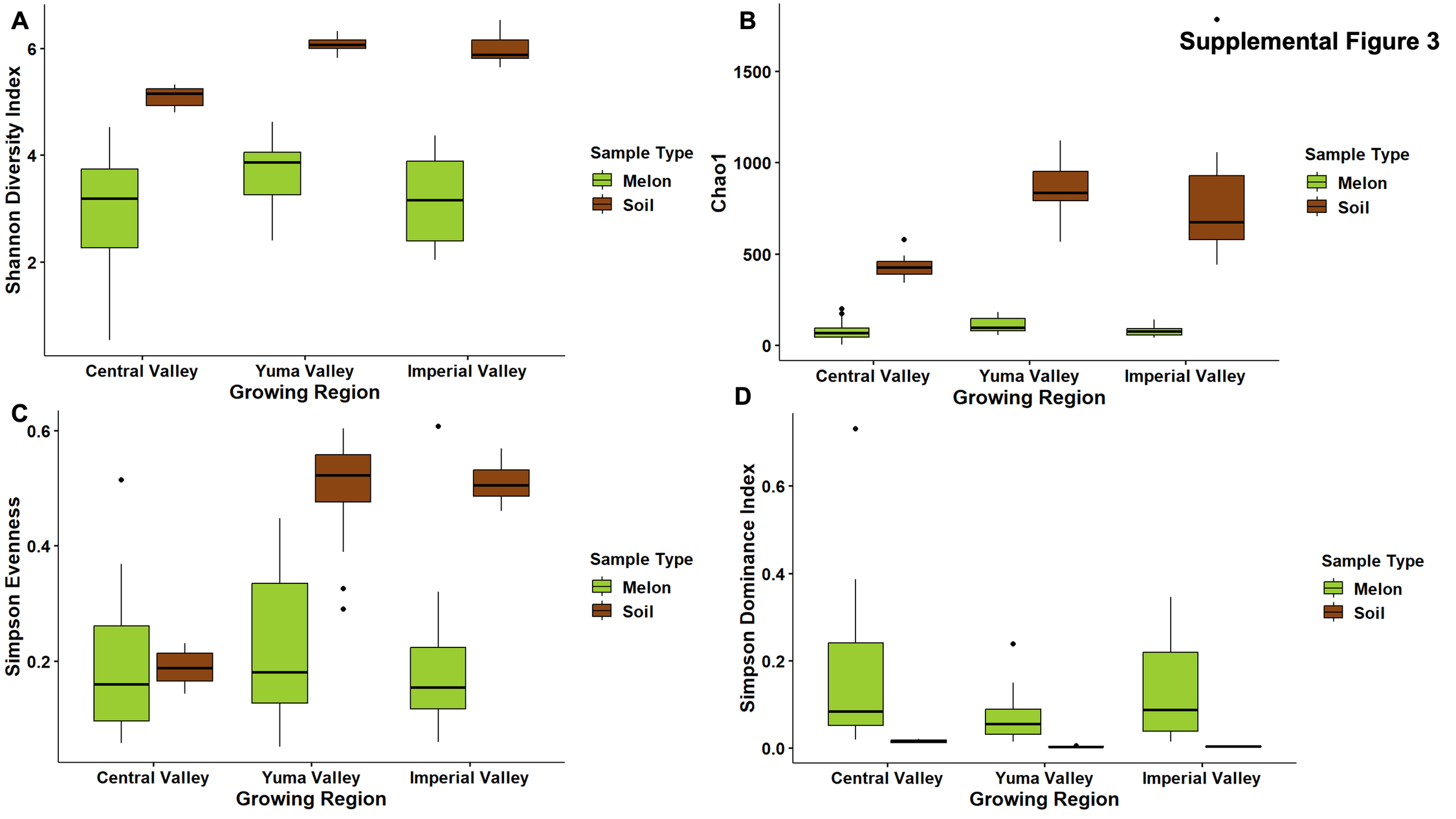

Supplement: S3 Fig — (A) Shannon Diversity Index plotted based on the site for the soil and melon samples considering both richness and evenness. (B) Chao1 total richness plotted based on the site for the soil and melon samples considering total taxonomic ASV richness. (C) Simpson Evenness plotted based on the site for the soil and melon samples considering distribution (evenness) of the bacteria across the samples. (D) Simpson Dominance Index plotted based on the site for the soil and melon samples considering inverse richness. (TIF) [file pone.0307477.s003.tif]
