# Supplementary material for: Bacterial diversity of cantaloupes and soil from Arizona and California commercial fields at the point of harvest
Source: PLoS One. 2024 Sep 26;19(9):e0307477. doi: 10.1371/journal.pone.0307477 (PMC11426484; doi:10.1371/journal.pone.0307477)
Supplement: S1 File — Supplemental information providing all the statistical tests used, parameters used for each statistical test, and the resulting p-values for each test that was run on all the data used in the study. (DOCX) [file pone.0307477.s004.docx]

Alpha matrices

| Alpha Shannon diversity | Kruskal Wallis | Wilcoxon |
| --- | --- | --- |
| Soil (of sites on Shannon) | 4.12x10^-3^ | Central to imperial: 8.4x10^-3^  Central to Yuma: 4.4x10^-5^  Imperial to Yuma: 1.00 |
| Soil with Yuma by month | 0.0025 | 0.002 (June to November) |
| Melons | 0.033 | Central to imperial: 1.00  Central to Yuma: 0.025  Imperial to Yuma: 0.583 |
| Soil-melons | 2.2x10^-16^ | 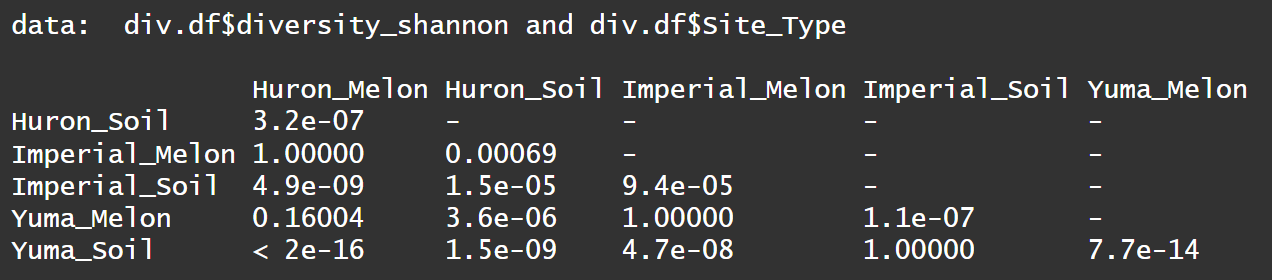 |

| Alpha Simpson Evenness | Kruskal Wallis | Wilcoxon |
| --- | --- | --- |
| Soil (of sites on Shannon) | 2.86x10^-3^ | Central to imperial: 8.4x10^-3^  Central to Yuma: 4.4x10^-5^  Imperial to Yuma: 1.00 |
| Soil with Yuma by month | 0.8816 | 0.89 |
| Melons | 0.6687 | Central to imperial: 1.00  Central to Yuma: 1.00  Imperial to Yuma: 1.00 |
| Soil-melons | 6.983x10^-8^ | 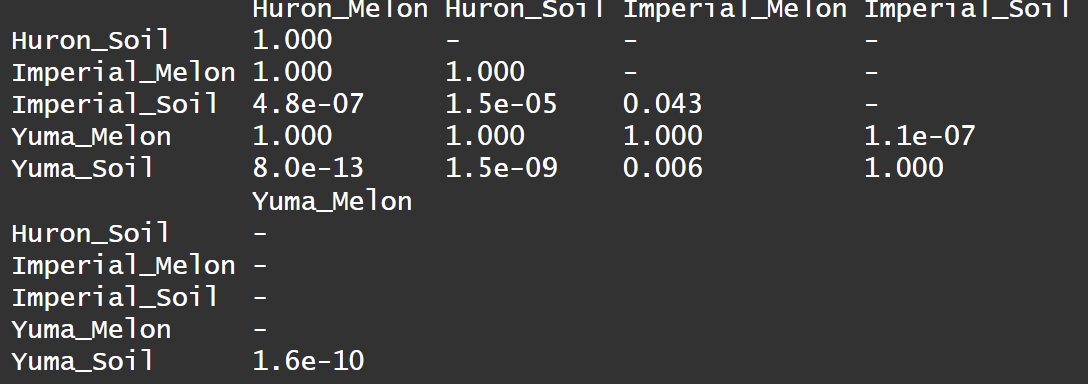 |

| Alpha Simpson Dominance | Kruskal Wallis | Wilcoxon |
| --- | --- | --- |
| Soil (of sites on Shannon) | 4.26x10^-3^ | Central to imperial: 8.4x10^-3^  Central to Yuma: 4.4x10^-5^  Imperial to Yuma: 1.00 |
| Soil with Yuma by month | 0.1473 | 0.15 |
| Melons | 0.089 | Central to imperial: 1.00  Central to Yuma: 0.089  Imperial to Yuma: 0.714 |
| Soil-melons | 4.969x10^-8^ | 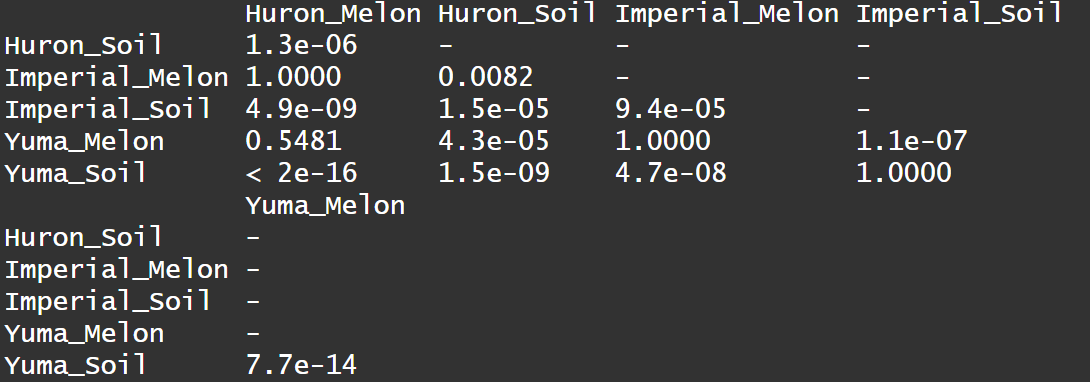 |

| Alpha Chao1 | Kruskal Wallis | Wilcoxon |
| --- | --- | --- |
| Soil (of sites on Shannon) | 4.22x10^-3^ | Central to imperial: 8.4x10^-3^  Central to Yuma: 4.4x10^-5^  Imperial to Yuma: 1.00 |
| Soil with Yuma by month | 1.76x10^-4^ | 8.3x10^-5^ |
| Melons | 0.03896 | Central to imperial: 1.00  Central to Yuma: 0.048  Imperial to Yuma: 0.257 |
| Soil-melons | 2.66x10^-8^ | 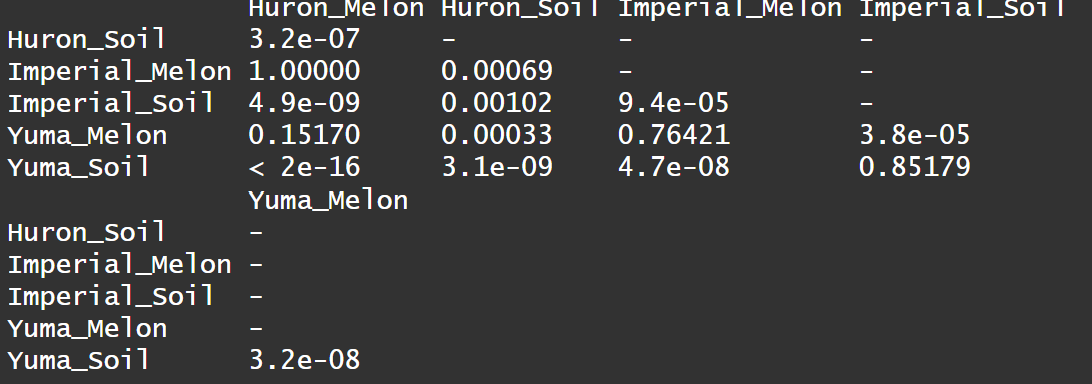 |
