# Supplementary material for: Bacterial diversity of cantaloupes and soil from Arizona and California commercial fields at the point of harvest
Source: PLoS One. 2024 Sep 26;19(9):e0307477. doi: 10.1371/journal.pone.0307477 (PMC11426484; doi:10.1371/journal.pone.0307477)
Supplement: S2 File — Includes a total of nine supplemental tables including: S1 Table. Reads per sample type across rarefaction limits; S2 Table. Core taxa shared among all regions for soil samples; S3 Table. Core taxa found in Yuma Valley for soil samples; S4 Table. Core taxa found in Imperial Valley for soil samples; S5 Table. Core taxa found in Central Valley for soil samples; S6 Table. Core taxon in all regions for melon samples; S7 Table. Core taxa found in Central Valley for melon samples; S8 Table. Core taxa found in Imperial Valley for melon samples; S9 Table. Core taxa found in Yuma Valley for melon samples. (DOCX) [file pone.0307477.s005.docx]

**Supplemental Table S1.** Reads per sample type across rarefaction limits

|  | **Range of Reads** | **Average Reads per Sample** | **Average Reads per Sample**  **(Post-filtering)** | **Rarefaction limit** | **Median # of Taxa (prior to rarefying)** | **Median # of Taxa (post-rarefying** |
| --- | --- | --- | --- | --- | --- | --- |
| Melons | 201-35,332 | 4,081 | 3,949 | 1,500 | 2,150 | 1,820 |
| Soil | 13,935-175,179 | 34,907 | 33,215 | 20,000 | 32,329 | 10,155 |
| Melons & Soil | 201-175,179 | 19,268 | 18,367 | 1,500 | 14,268 | 8,102 |

**Supplemental Table S2.** Core taxa shared among all regions for soil samples.

|  | Family | |
| --- | --- | --- |
| Regions: Yuma, Imperial, & Central Valley | *Gemm-1**  *Gemmatimonandetes**  *envOPS12***  *JG30-KF-CM45***  *MND1***  *iii1-15***  *Bacillaceae*  *Bradyrhizobiaceae*  *Comamonadaceae*  *Chitinophagaceae*  *Cystobacteraceae*  *Gaiellaceae* | *mb2424*  *Micrococcaceae*  *Nitrosomonadaceae*  *Nitrososphaeraceae*  *Nitrospiraceae*  *Nocardioidaceae*  *Oxalobacteraceae*  *Piscirickettsiaceae*  *Planococcaceae*  *Rhodospirillaceae*  *Sinobacteraceae*  *Syntrophobacteraceae* |

*: At the Class Level

**: At the Order Level

**Supplemental Table S3.** Core taxa found in Yuma Valley for soil samples

|  | Family | |
| --- | --- | --- |
| Region: Yuma Valley | *Gemmatimonadetes*^,^***  *Acidobacteria-6***  *Gemm-1***  *envOPS12****  *JG30-KF-CM45****  *Rhizobiales****  *MND1****  *Ellin6067****  *iii1-15****  *Bacillaceae*  *Bradyrhizobiaceae*  *Chitinophagaceae*  *Comamonadaceae*  *Cystobacteraceae*  *Cytophagaceae* | *Gaiellaceae*  *mb2424*  *Micrococcaceae*  *Nitrosomonadaceae*  *Nitrososphaeraceae*  *Nitrospiraceae*  *Nocardioidaceae*  *Oxalobacteraceae*  *Piscirickettsiaceae*  *Planococcaceae*  *Rhodospirillaceae*  *Sinobacteraceae*  *Syntrophobacteraceae*  *Xanthomonadaceae* |

*: At the Phylum Level

**: At the Class level

***: At the Order Level

**Supplemental Table S4.** Core taxa found in Imperial Valley for soil samples

|  | Family | |
| --- | --- | --- |
| Region: Imperial Valley | *C0119**  *Ellin6529**  *Gemmatimonadetes**  *Gemm-5**  *WD2101***  *Solibacterales***  *S0208***  *envOPS12***  *JG30-KF-CM45***  *Rhodospirillales***  *MND1***  *RB41***  *iii1-15***  *Solirubrobacterales***  *0319-7L14***  *0319-6A21*  *A4b*  *AKIW874*  *Bacillaceae*  *Bradyrhizobiaceae*  *Chitinophagaceae*  *Clostridiaceae*  *Comamonadaceae*  *Cystobacteraceae* | *Ellin6075*  *Euzebyaceae*  *Gaiellaceae*  *Kineosporiaceae*  *mb2424*  *Methylobacteriaceae*  *Micrococcaceae*  *Micromonosporaceae*  *Mycobacteriaceae*  *Nitrosomonadaceae*  *Nitrososphaeraceae*  *Nitrospiraceae*  *Nocardioidaceae*  *Paenibacillaceae*  *Pirellulaceae*  *Planococcaceae*  *Rhodospirillaceae*  *Rhodothermaceae*  *Rubrobacteraceae*  *Sinobacteraceae*  *Sphingomonadaceae*  *Streptomycetaceae*  *Syntrophobacteraceae* |

*: At the Class Level

**: At the Order Level

**Supplemental Table S5.** Core taxa found in Central Valley for soil samples

|  | Family | |
| --- | --- | --- |
| Region: Central Valley | *Ellin6529**  *Actinobacteria**  *Gemm-1**  *Solibacterales***  *AKYG1722***  *JG30-KF-CM45***  *Solirubrobacterales***  *Iii1-15***  *0319-6A21*  *Bacillaceae*  *Beutenbergiaceae*  *Bradyrhizobiaceae*  *Caldilineaceae*  *Caulobacteraceae*  *Cystobacteraceae*  *Cytophagaceae*  *Dolo_23*  *Gaiellaceae*  *Geodermatophilaceae*  *Hyphomicrobiaceae*  *mb2424*  *Microbacteriaceae* | *Micrococcaceae*  *Micromonosporaceae*  *Mycobacteriaceae*  *Nitrosomonadaceae*  *Nitrososphaeraceae*  *Nitrospiraceae*  *Nocardioidaceae*  *Oxalobacteraceae*  *Planococcaceae*  *Promicromonosporaceae*  *Pseudomonadaceae*  *Rhizobiaceae*  *Rhodospirillaceae*  *Sinobacteraceae*  *Solirubrobacteraceae*  *Sphingobacteriaceae*  *Streptomycetaceae*  *Streptosporangiaceae*  *Syntrophobacteraceae*  *Thermomonosporaceae*  *Xanthomonadaceae* |

*: At the Class Level

**: At the Order Level

**Supplemental Table S6.** Core taxon found in all regions for melon samples

|  | Family |
| --- | --- |
| Regions: Yuma, Imperial, & Central Valley | *Micrococcaceae* |

**Supplemental Table S7.** Core taxa found in Central Valley for melon samples

|  | Family |
| --- | --- |
| Region: Central Valley | *Bacillaceae*  *Enterobacteriaceae*  *Geodermatophilaceae*  *Micrococcaceae* |

**Supplemental Table S8.** Core taxa found in Imperial Valley for melon samples

|  | Family |
| --- | --- |
| Region: Imperial Valley | *Bacillaceae*  *Peptostreptococcaceae*  Turcibacteraceae |

**Supplemental Table S9.** Core taxa found in Yuma Valley for melon samples

|  | Family |
| --- | --- |
| Region: Yuma Valley | *[Exiguobacteraceae]*  *Micrococcaceae*  Planococcaceae |
