# Supplementary material for: Bacterial diversity of cantaloupes and soil from Arizona and California commercial fields at the point of harvest
Source: PLoS One. 2024 Sep 26;19(9):e0307477. doi: 10.1371/journal.pone.0307477 (PMC11426484; doi:10.1371/journal.pone.0307477)
Supplement: Attachment — Submitted filename: Revision Notes.docx [file pone.0307477.s007.docx]

Reviewer Comments

**General Comments**

This manuscript describes the characterization of the microbiome from the surface of cantaloupe rinds and soil from cantaloupe growing regions. The manuscript is improved and many of my concerns have been addressed. Specific minor edits needed are listed below.

**Specific comments**

Lines 49 - 50: Change “community taxonomic constitutions” to “community structure”

Line 58: Replace “had” with “identified”

Line 79: Replace “eaten” with “produced”

Lines 95 – 97: This sentence is very confusing. Please reword for clarity.

Line 113: Consider replacing “However, it is” with “Given these risks,”

Line 123: replace “consumerism” with “consumption”

Line 124 - 125: This sentence is awkward as written. Perhaps replace “kept on a flotation device” with “keeping melons on a flotation device”

Line 126: Delete “However”

Line 135: Replace “at” with “where there are”

Line 162: Replace “generated by collecting samples” with “collected”

Line 171 - 172: Rewrite this sentence as “DNA was extracted from each composite soil sample and cantaloupe swab using a Qiagen…”

Line 203: “Q30 forward” should be “Q30 for forward”

Line 221 and 223: Replace “left” with “retained”

Line 222: Replace “of” with “from”

Line 227: Rewrite as “phyloseq, microbiome, and microbiomeutilities R packages [26, 29]”

Lines 232 – 234: This sentence is rather nonsensical and needs to be rewritten.

Line 239: What was the justifications for using a 75% prevalence threshold for defining the core microbiome?

Lines 241 – 243: This sentence is poorly written and unclear. Please rewrite.

Line 244: what is meant by “parameter of relative detection at 1x10^-9^”?

Line 246 – 247: This description isn’t very clear. LEfSe is used to determine the features most likely to explain differences between classes. Please clarify how this is being used and the classes that are being compared.

Line 247: replace “have been” with “were”.

Line 264: replace “found” with “showed”

Lines 281 – 283: Please clarify. The core was defined in the methods as 75% prevalence, 0.1% abundance. Is it now being redefined with a 1% abundance and if so, why?

Lines 323 – 327: This should be moved to the Discussion.

Line 372: missing “p-value” in front of (4.7x10^-8^).

Line 404: “in melon core” should be “in the melon core”

Line 407: replace “This also identified” with “These results indicated”

Lines 437 – 440: Rewrite this sentence. For example, what does “using different land applications” mean?

Line 446: Replace “away” with “separate”

Line 463: Replace “that” with “a”

Line 471: For clarity, rewrite “*Bacillus,* from *Bacillaceae,*” as “*Bacillus*, a genus in the family *Bacillaceae*,”

Lines 506 – 509: Reword this sentence. How do immature melons play a role in the family *Enterobacteriaceae*?

Line 535: “nearly not completely impossible” ???

Line 544: Delete “Yet”

Line 556 Delete “e”

Line 564: delete extra period
